# Is It Possible to Monetarily Quantify the Emotional Value Transferred by Companies and Organizations? An Emotional Accounting Proposal

**DOI:** 10.3389/fpsyg.2021.805920

**Published:** 2022-01-04

**Authors:** Jose Luis Retolaza, Leire San-Jose

**Affiliations:** ^1^HUME, Deusto Business School, University of Deusto, Bilbao, Spain; ^2^ECRI, Financial Economics II, University of the Basque Country, Bilbao, Spain

**Keywords:** utility, emotional value, stakeholder, monetize, monetization, social impact, social value, social accounting

## Abstract

Social accounting focuses on value transactions between organizations and their stakeholders; both market ones, where the value perceived by the different stakeholders is identified, and non-markets ones, where transactions are monetized at their fair value. There was long awareness of an emotional value translation, linked to the transfer of different products, services, remunerations, and incentives, regardless of whether they were market or non-market. Yet that emotional value seemed to be anchored in the field of psychology and managed to elude economic science. This study seeks to identify emotional value with consumer surplus and, by extension, of the other stakeholders in a value transfer process. This proposal allows the emotional value to be anchored in the micro-economy and allows it to be objectively calculated using a regression involving three elements: the market price, the fair value interval, and a perceived satisfaction score by the different stakeholders in the form of significant sampling. The result obtained not only allows Social Accounting to be complemented with emotional value, but it also facilitates its incorporation in the strategy to optimize the emotional value. Furthermore, it enables a quantification of the perceived subjective utility, which opens up a research path where some possible lines are clearly identified.

“*Uncritical enthusiasm for mathematical formulation tends often to conceal the ephemeral substantive content of the argument behind the formidable front of algebraic signs”* Wassily Leontief

## Introduction

In recent years, the breakdown of the microeconomic model relating social optimum to profit maximization has led to the development of complementary social accounting that is capable of measuring, preferably in economic terms, the value generated and distributed to the stakeholders. In general, the accounting incorporates the market value, through economic-financial accounting, and the non-market value, through the fair value. In turn, although the transferred emotional value is considered a fundamental element, it has so far remained elusive to a systemic attempt to its measuring in monetary units. This study seeks to develop a proposal to allow a systematic monetary valuation of that type of value, based on the supply and demand curve.

Although the work is theoretical, it is underpinned by a large number of cases analyzed in recent years by the social accounting for sustainability (SAS) research group, recognized by International Centre of Research and Information on the Public, Social and Cooperative Economy (CIRIEC), some of which are published in leading journals, general papers (Retolaza et al., 2016; Retolaza and San-Jose, 2021), on its application in the social economy in general (Lazcano et al., 2019; Lazkano and Beraza, 2019; Etxanobe, 2020; Lazkano et al., 2020), on its usefulness to measure hospital efficiency (San-Jose et al., 2019), about sport clubs (Mendizabal et al., 2020; Mendizabal and Garcia-Merino, 2021), applied to ecclesiastical organizations (Retolaza et al., 2020), to universities and education (Ayuso et al., 2020; Arimany-Serrat and Tarrats-Pons, 2021; Barba-Sánchez et al., 2021a), its benefit for technology parks (Blázquez et al., 2020; Torres-Pruñonosa et al., 2020), associations of fishers (Guzmán-Pérez et al., 2018), tourism (Guzmán-Pérez et al., 2021), public tenders (Bernal et al., 2019), and agri-food companies (Barba-Sánchez et al., 2021b). These studies identify the importance of emotional value and propose its inclusion in social accounting, which has so far been partially addressed by few studies (Ruiz-Roqueñi, 2020; Tirado-Valencia et al., 2021).

Therefore, this study presents a system to quantify the emotional value. For this purpose, in the second section, the theoretical framework is shown, and after that, the proposal for quantifying the emotional value on organizations, the range of the value, extension to whole stakeholders, degrees of satisfaction, and the full process are explained. The study ends with a discussion and concluding remarks, limitations, and future research.

## Theoretical Framework

Nearly three centuries ago, Bernoulli (1738), building on his well-known St. Petersburg paradox, introduced the concept of expected utility to incorporate aspects such as morality and emotions into the decision process, which solved, at least in theory, the constraints of the classic paradigm regarding excepted value. The first notion of expected utility was subsequently formalized by Von Neumann and Morgenstern (1947) and developed in the field of game theory. If people maximize something unknown called utility instead of maximizing the expected value, many decisions that would otherwise be considered irrational could be explained. In this vein, the subsequent reflection has fundamentally focused on decision-making linked to uncertainty processes and not to such an extent on the morality and emotional sphere that would be opened by reconceptualization (see Table 1).

**Table 1:** Example of net promoter score.

Market price (breakeven)	α	€15
Upper limit of the fair value	p_max_	€25
Lower limit of the fair value	p_min_	€10
S*_*i*_* X*_*i*_* ≥ 5 → [p_max_ – p_m_]; S*_*i*_* X*_*i*_* ≤ 5 → [p_min_ – p_m_]	β	+10
Satisfaction score (NPS), *i* = 1–10	Xi	8.05

The origin of expected utility emerged from the need to solve the problem of the actual behavior of people not being fully rational, at least in economic terms. If, on the contrary, an individual subjectivism is accepted of the profit-loss binomial about such aspects as emotions, morals, or uncertainty, an irrational choice from the point of view of the economic value may be fully rational from the utility expected by the specific individuals. From this perspective, the expected utility introduced subjective value elements, complementing the economic value objectified in the price. Subsequently, Kahneman and Tversky (1979) noted that the principles proposed by Bernoulli are mathematically valid, but poorly explained actual human behavior, which is clearly affected by cognitive processes that distance it from the rational man ideal underlying the expected utility theory. Simon (1972, 1979) had already pointed out that people do not act as profit maximizers but rather as satisficers. Similarly, Samuelson (1993) defined “utility” as an expression of satisfaction; in other words, the subjective pleasure reported by a person after consuming a good or service.

In contrast, another appraisal must be made. During nearly three centuries of economic reflection following the introduction of the concept, and despite the specific contribution of the economic behavior, a model has not been proposed that supports the monetary quantification in the form of individual assessment, which at the end of the day runs the risk of turning it into a tautological concept. A payment is worth so much, if the payment is what it is worth, at least for me; given that that specific value for each person cannot be identified, any payment adequately reflects consumer preferences and is, therefore, a rational choice; yet totally indisputable, as we cannot find examples of irrational conducts, and, therefore, beyond scientific knowledge.

Being able to identify what the standard value (breakeven price) of a good or service is and the value of the set of attributes accompanying that good or service is for a specific consumer could greatly help to advance in researching the idiosyncratic utility. This can be defined as the difference between the expected value and the expected utility, where the first concept is understood as a purely economic concept, whereas the second concept incorporates the complementary aspects that can be valued by each specific individual, and this is called as a differential emotional value.

$$\begin{array}{l} \text{Emotional value}=\text{Expected utility}-\text{Expected value}\\ \text{ Ę}(x)=\text{u}\left(x\right)-\text{E}\left(x\right) \end{array}$$

Hence, the emotional value would materialize as an increase or decrease in the expected economic value, represented by the breakeven point between supply and demand.

$$\begin{array}{l} \text{Emotional value}=\text{Increase}/\text{decrease of the market}\\ \text{ value }\left(\text{price}\right)\;\\ \text{ Ę}(x)=\text{ŦE}\left(x\right) \end{array}$$

From this perspective, the emotional value functions as a correction factor upward or downward of the value identified by the price, in the case of the market transactions, or by the fair value, in the case of non-market transactions. Given that we already know the economic value of the transfer, two complementary data would be needed to quantify monetarily the perceived emotional value: the degree of satisfaction of the set of recipients and the scope of the value range.

Regarding the first question, given the nature of the expected utility, there is no other option than to determine the average value using a representative sample of recipients. It should be noted that, as the recipients are distributed into interest groups, the significance of the sample should be maintained for each of the groups of stakeholders, at least to identify not only the consolidated emotional value but also the specific distribution to each of the groups of stakeholders. The solution for this first point may be painstaking, but theoretically it is not complicated.

The second point is much more complicated to address. We know the breakeven economic value and, therefore, we can consider that, if the satisfaction is correctly measured, its breakeven point (neither satisfied nor dissatisfied) would tally with that breakeven value. In other words, null satisfaction (neither positive nor negative) would not add any emotional value to the breakeven value that we already have. Therefore, there would be parity between the expected value and the expected utility:

$$\begin{array}{l} \text{ Ę}(x)\to \text{u}\left(x\right)=\text{E}\left(x\right);\\ \text{or its inverse}:\text{ u}\left(x\right)=\text{E}\left(x\right)\to \text{Ę}(x) \end{array}$$

Yet if the satisfaction score shifts toward the minus or plus, we cannot determine the monetary value or that addition or subtraction as the maximum and minimum point of the perceived utility is not known. A simple solution consists of establishing a fair value range, using expert consensus or another technique, which was followed in the early research in this respect (Ruiz-Roqueñi, 2020), where a range of ±50% was established. However, the solution has some theoretical and practical problems. In the first case, there is a great deal of research that suggests that people are not symmetrical in their utility assessment (Kahneman and Tversky, 1972) and express asymmetrical expectations regarding the assumed risk, as in the case of wishful thinking (Babad and Katz, 1991), which would mean that the (+) and the (–) would be asymmetrical. In contrast, we came across value variables that would clearly exceed ±50%, e.g., in the world of sport (Mendizabal and Garcia-Merino, 2021), where the emotional value may be much greater than the economic value *per se* generated using transfer mechanisms, regardless of whether they are market or non-market.

To obtain a monetary calculation of the emotional value generated for the different stakeholders and, by consolidation, for society, we must address and diligently settle the two previously indicated aspects, namely, (1) the mechanism to measure stakeholder satisfaction and (2) the range of values around the breakeven point, identified by the price or the fair value. The following sections address both challenges in reverse order to how they are listed above.

## Qualifying the Range of the Monetized Emotional Value

**Figure 1:**
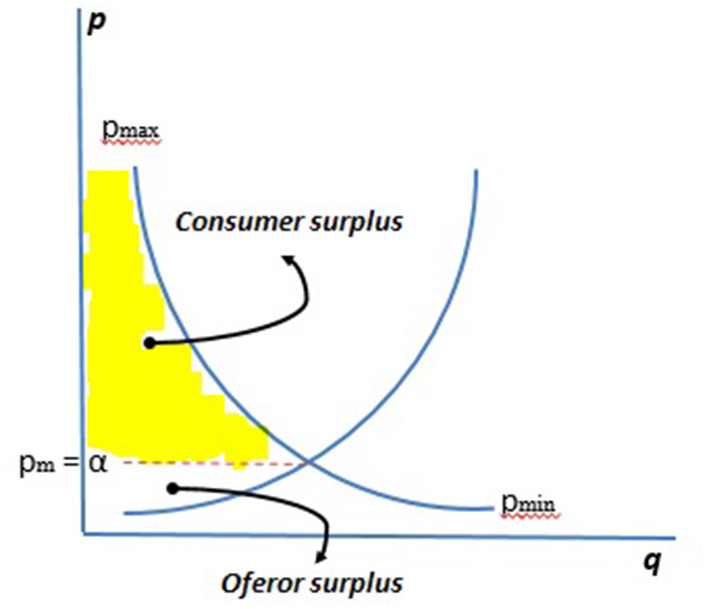
Range qualifying demonstration. Source: own compilation based on Samuelson and Nordhaus (1989).

The supply curve and demand curve are an established and accepted logic framework in economics. It can, therefore, be a starting point for understanding and solving the problem of the emotional value range. As is well-known, both curves cross at the breakeven point, which reflects the price of a service or product for a certain market. The breakeven point acts as the base to calculate the surplus of the consumer and of the producer, taken to be the difference between the total utility obtained from the purchase or sale of a good or service and its market price. In other words, the difference between the value and the utility (see Figure 1).


$$\begin{array}{l} \text{Consumer surplus }=\text{Maximum price willing to pay}\\ \;-\text{the real price} \end{array}$$

Taking into account that the maximum price willing to be paid finds its upper limit in the marginal utility obtained, the aforementioned parity corresponds to:

$$\begin{array}{l} {\text{E}}_{\text{c}}\left(x\right)=\text{U}\left(x\right)-\text{E}\left(x\right) \end{array}$$

Which, as can be seen, is equivalent to the formula defined for the emotional value, which when aggregated would tally with the consumer surplus.

$$\begin{array}{l} \text{Ę}(x)=\text{U}\left(x\right)-\text{E}\left(x\right)\approx {\text{E}}_{\text{c}}\left(x\right) \end{array}$$

Therefore, for its individual calculation, it can be considered that

$$\begin{array}{l} \text{Ę}(x)\approx {\text{E}}_{\text{c}}\left(x\right)=\left[{\text{p}}_{\text{max}}\left(x\right)-{\text{p}}_{\text{m}}\right]{\;}^{*}\;\beta \end{array}$$

β is the perceived utility level

While it can be calculated globally as:

$$\begin{array}{l} \text{Ę}(x)\approx {\text{E}}_{\text{c}}\left(x\right)=\left[{\text{p}}_{\text{max}}\left(x\right)-{\text{p}}_{\text{m}}\right]{\;}^{*}\;\beta_{\text{i}} \end{array}$$

*i* is the (1, …, *n*) utility perceived by *n* number of individuals

**Figure 2:**
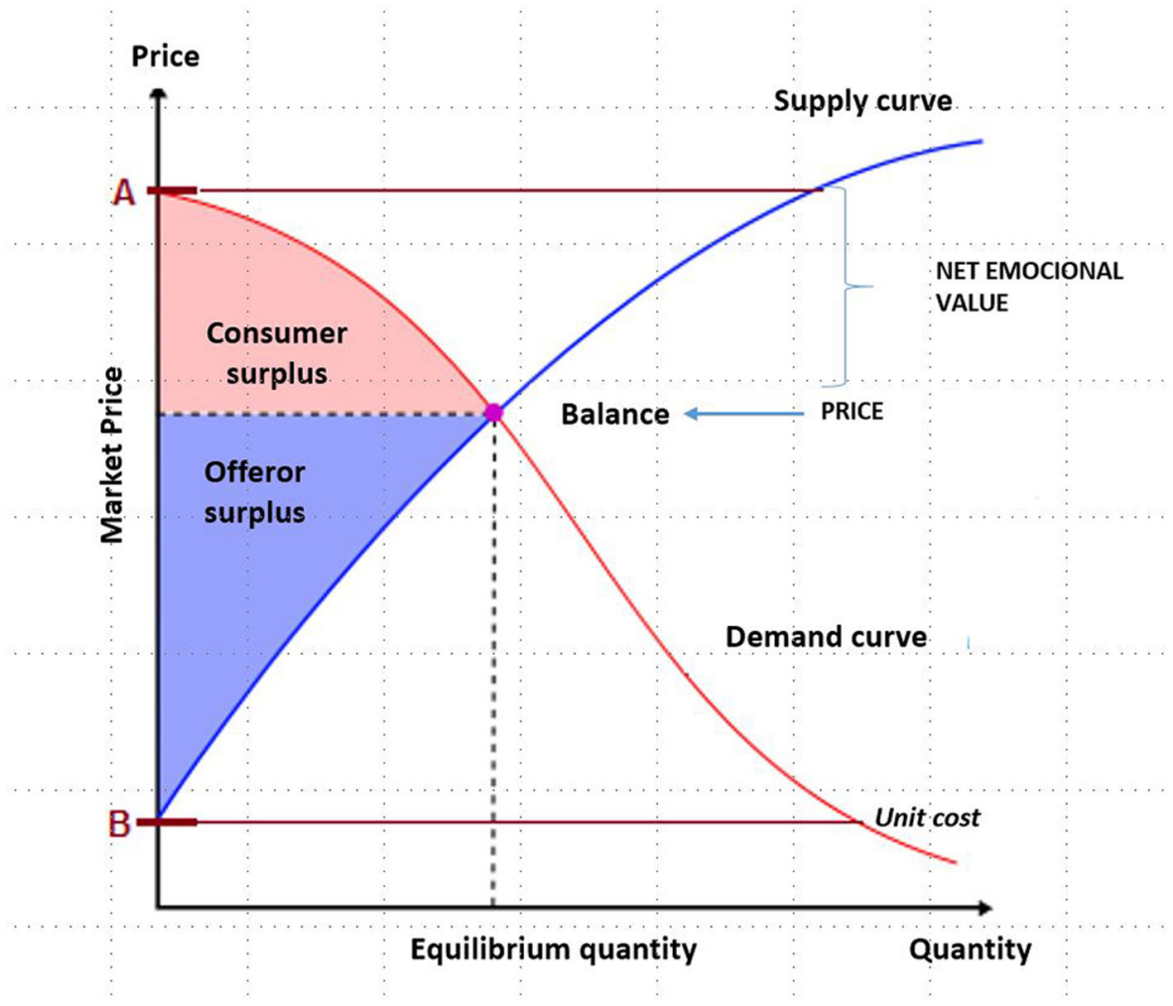
Emotional value breakeven point. Source: own compilation based on Samuelson and Nordhaus (1989).

**Figure 3:**
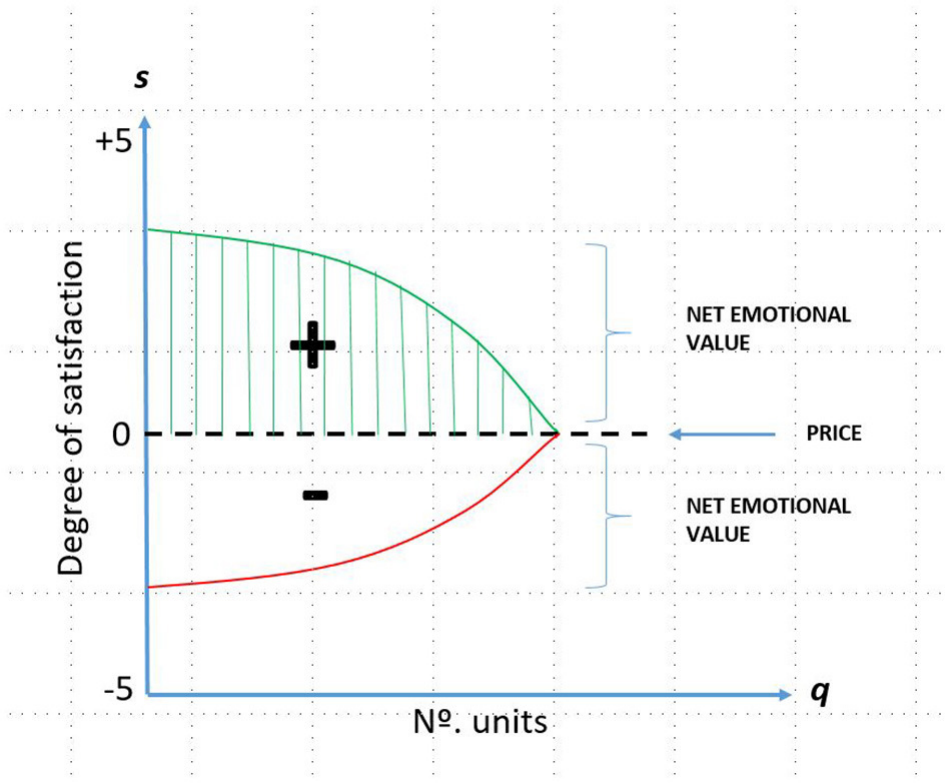
Net emotional value price. Source: own compilation based on Samuelson and Nordhaus (1989).

Figures 2, 3 visually depict the emotional value considered as the difference between the value and the utility and, therefore, represented by the area generated by the breakeven price and the maximum willingness to pay multiplied by the number of units.

However, the emotional value is identified as the difference between what the consumer would be willing to pay for the product or service received and what they have actually paid for it. As can be deduced, the emotional value can be positive and negative, in those cases where the perceived utility after the purchase of the good or service is lower than the purchase price (market value) (Figure 3).

**Figure 4:**
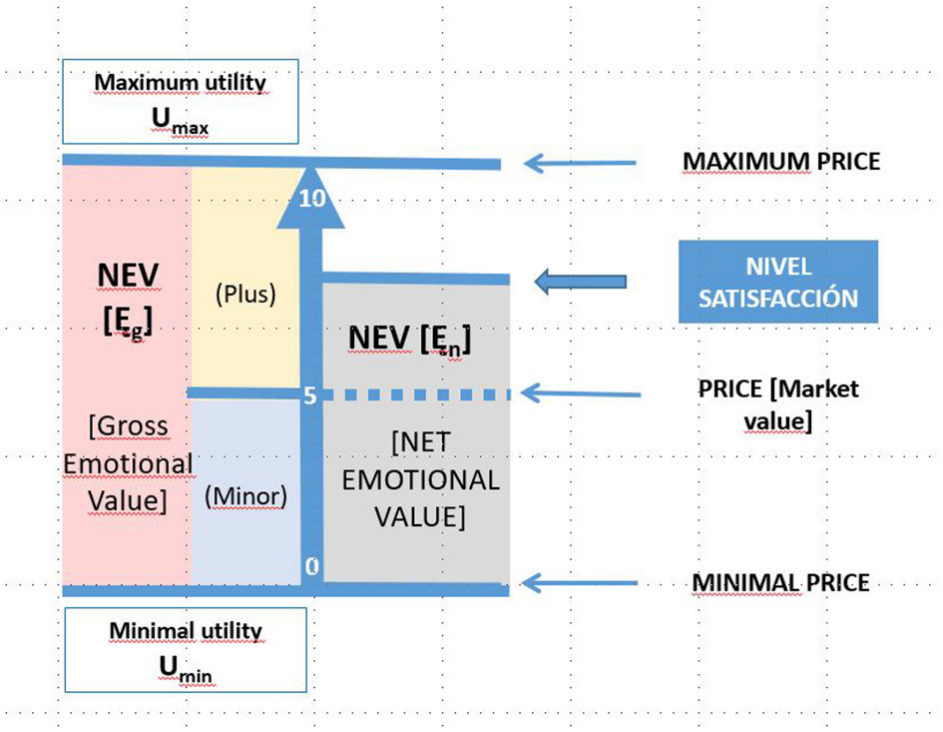
Net emotional value. Source: own compilation. (1) The numbers in white located on the horizontal axis are only decimal references to make the information easier to see; under no circumstances should it be assumed that the percentages of each value must be related to them. (2) Should the price not match the market value due to market failure, the appropriate corrections would have to be applied.

Figure 4 shows the relationship between the different costs and value types generated in a transactional market relationship between supplier and recipient. As can be seen, the potential emotional value [E_g_ = GEV] is taken to be the maximum that could be generated and corresponds to the perceived maximum utility, i.e., with the maximum willingness to pay by the recipients, and would correspond to the perceived utility. We refer to this emotional value as gross compared with the one actually obtained in a transaction, which is net emotional value [Ę = NEV]; the difference between the net and the gross represents the potential margin to improve the experience of consumers.

## Expanding the Emotional Value to the Set of Stakeholders

Despite the above analysis has been performed based on the interrelation of what has been consumed with the producer, it can be expanded to the set of stakeholders, or at least, those who interact with the organization in exchange for a payment. Although it is true that, in the case of suppliers and production factors, the organization acts as the recipient and the stakeholders as producers, a market value supposedly exists, which corresponds to the agreed price. If the price is subsidized, either totally or partially, the benchmark price would not be the benchmark fair value but rather the minimum value, represented by the lower end of the fair value interval.

There is also a market value in the case of the interrelation with the suppliers and stakeholders that can be considered as production factors, such as the workers or investors. In the case of suppliers, this value is expressed in the price. In the case of workers, it would be the salary, which has certain market restrictions, such as the minimum wage or collective agreements; it should be noted that the emotional salary concept is making its mark in this area. In the case of capital, a difference would have to be made between fixed-price investors, such as lenders, and shareholders, where the expected value is an expectation likely to be modified and where the risk margin plays an important role. Concerning other more general stakeholders such as residents or citizens, the market value may be calculated using the costs or the fair value of the outsourcing, whether negative or positive.

**Figure 5:**
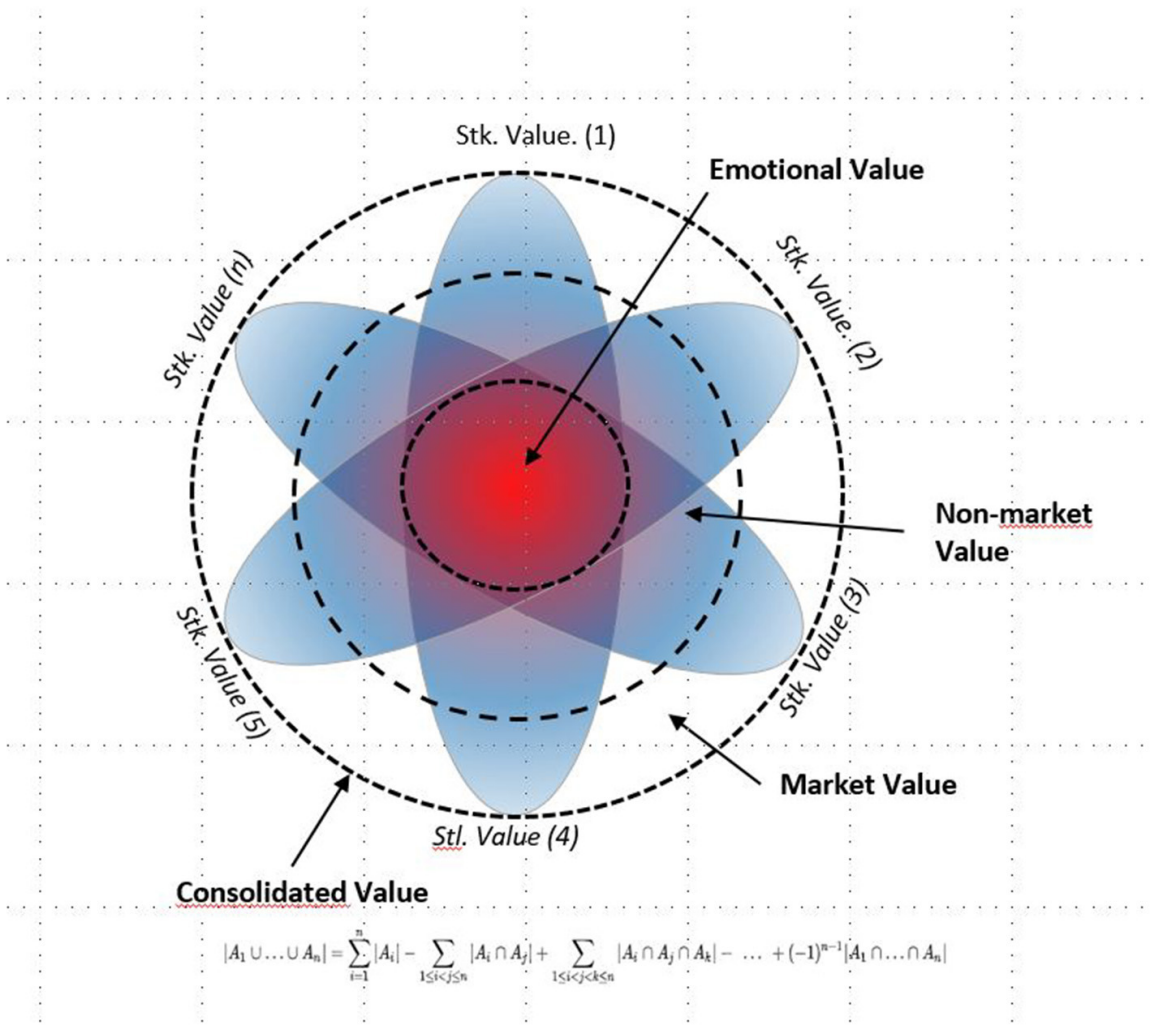
Emotional value in the multifaceted model. Source: Freeman et al. (2020, p. 102).

In any event, we find that each of the stakeholders interacts with the organization in an interplay between supply and demand, where the stakeholder adopts a similar role to that of the consumer and the organization to that of the producer, and a breakeven price is indeed generated that can be lower or higher to the perceived utility, regardless of the degree of freedom in setting the exchange price. Normally, the utility is greater than the value, otherwise the balance is unstable, except when the transaction costs are excessively high (see Figure 5).

Figure 5 shows the differential perception by the different stakeholders of each value type: market, non-market, and emotional. Note that the market value is exclusive in its appropriation, whereas the non-market value is partially shared. To a great extent, the emotional value is necessarily shared between the different stakeholders interacting in the transfer process.

## Discussion and Assessment of the Degree of Satisfaction of the Stakeholders

Unlike the rest of the emotional value, which can be inferred from the market, whether directly through trading processes or indirectly using the fair value when the transfer is not a market one, the transferred emotional value is a closely personal matter, in a similar way as regards utility. Accordingly, it cannot be calculated generically, as the difference between the price paid and the satisfaction obtained is specific for each individual. Therefore, it is necessary to have information on the population. In some cases, the population is limited, such as in the case of the workers of a micro-company, where it is possible to obtain information for the population as a whole; but it is not possible in other cases, for example, the customers of a retail area, and we have to resort to a significant sampling of the population.

**Figure 6:**
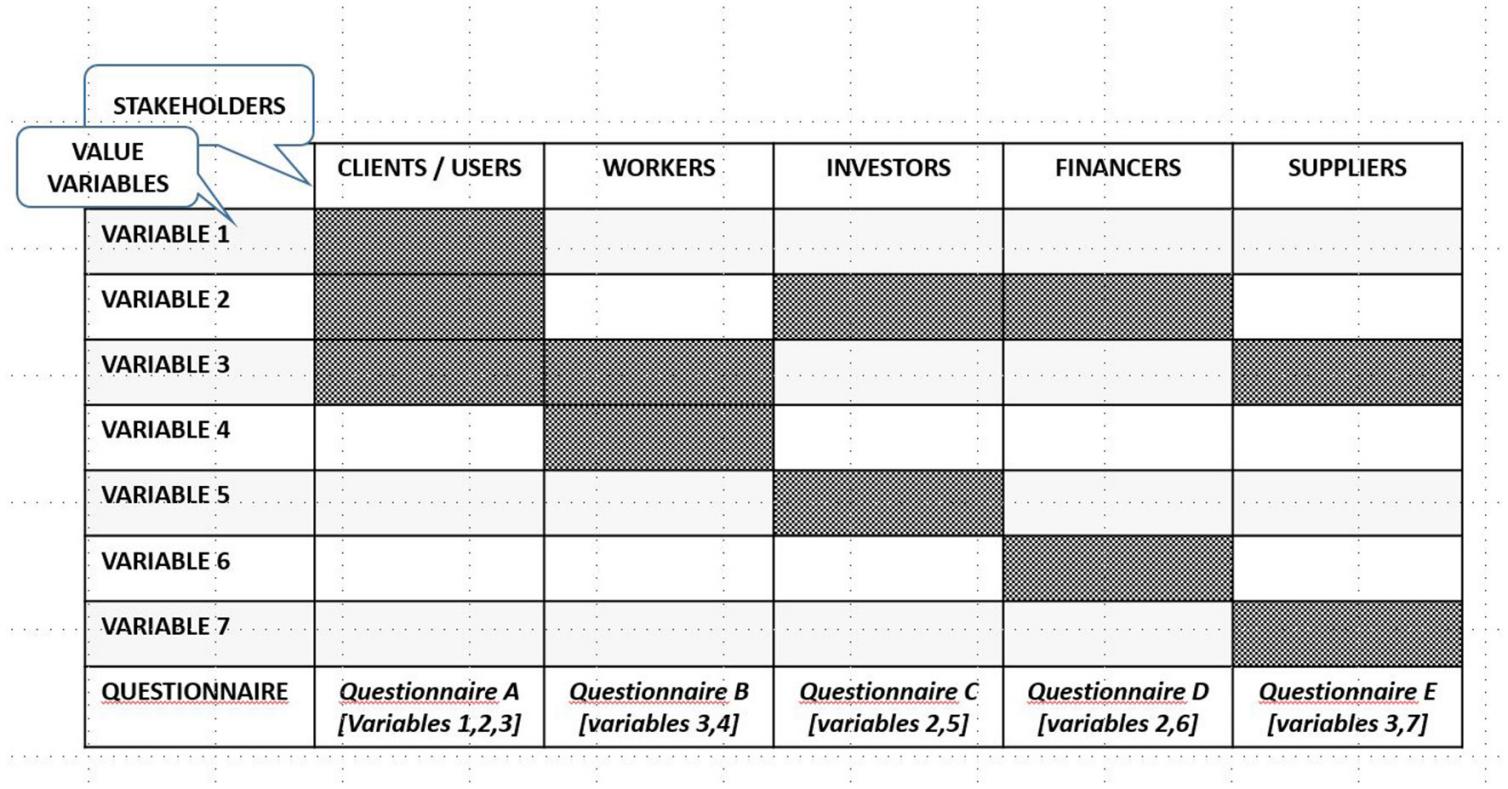
Matrix for the variable and stakeholder synthesis. Source: own compilation.

As it cannot be inferred whether the emotional value generated for the different stakeholders is uniform, significant sampling is needed for each of the groups whose emotional value should be analyzed. The most common ones are customers, workers, investors, funders, and suppliers. The questionnaire must also be adapted to the different stakeholders, as, in the process to identify the value variables, there may be some variables common to several groups but others would be specific. It seems reasonable for each group to be questioned about the variables through which the value is transferred. Therefore, the information gathered must consider two complementary dimensions: the stakeholder group and the different variables (see Figure 6).

Regarding the specific methodology to conduct the satisfaction assessment, it is a contingent matter and the selection can be performed using what is published in other fields of business administration, such as marketing or quality analysis. At the time of writing the article, and without it being an exclusive proposal, it seems that the net promoter score (NPS) could be a good option. It is a methodology that measures customer satisfaction through their loyalty, measured in terms of the probability of their recommending a product or service of the company. In the case of the emotional value, for obvious reasons, it would spread from the customer to other stakeholders and the value variables would be assimilated to products or services. The questionnaire consists of a single question, namely, what is the likelihood of you recommending the value variable (specifying which) to somebody else? This question would be multiplied by each of the value variables identified for each stakeholder. Additionally, another general question can be included for the question overall, such as, what is your current mood? That question could be used to perform a weighted adjustment of the direct scores obtained in the previous question.

The proposed formula to calculate the resulting score would be:

$$\begin{array}{l} \text{NPS}=\text{\%Promoters}-\text{\%detractors} \end{array}$$

**Figure 7:**
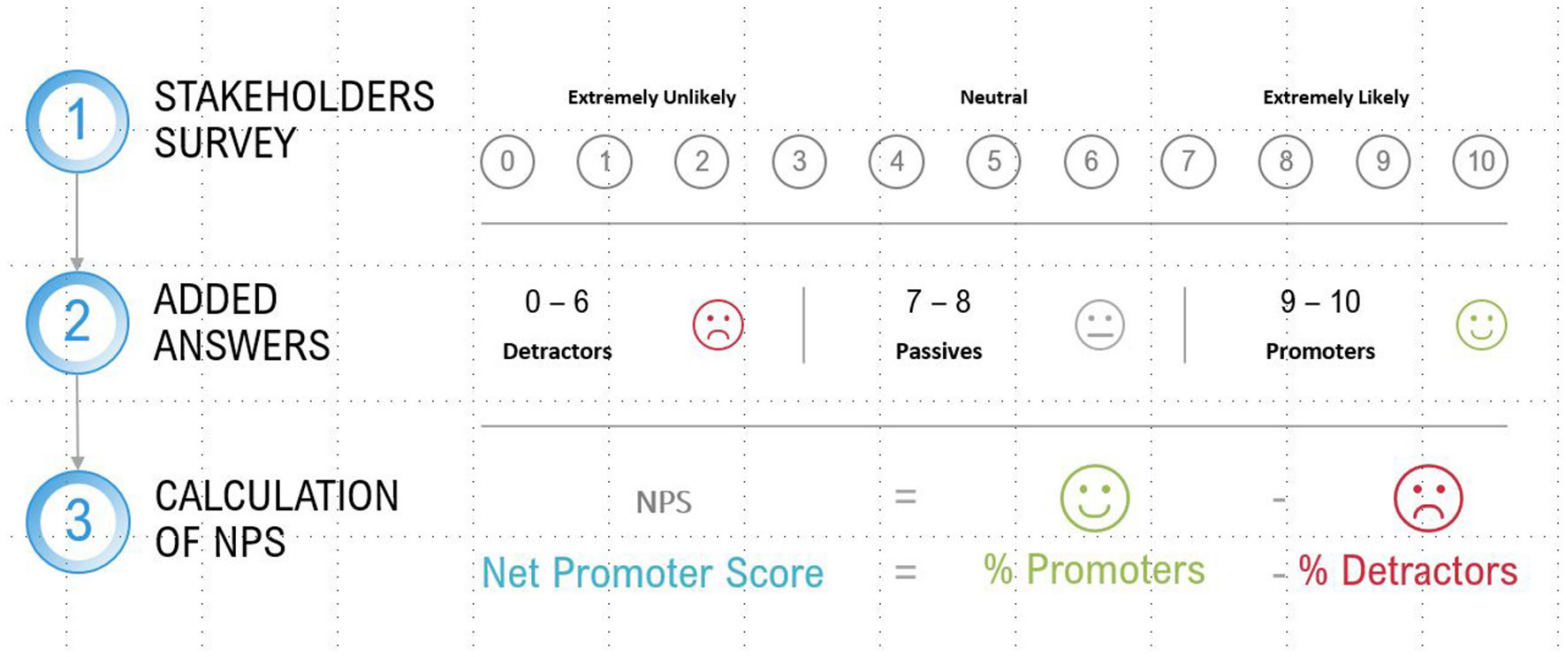
Representation of the net promoter score (NPS). Source: own compilation.

For this calculation, score of 9 or 10 is considered as promoters, and a score of 6 or below is considered as detractors. Scores 7 and 8 are considered neutral and are known as passives (see Figure 7).

It should be noted that the resulting NPS is for each variable, but not for the organization overall. Given that the value intervals of the different variables may be very different, the calculation must be performed for each specific variable and then the individual values obtained must be added up. Obtaining an average NPS and applying it to the different variables may lead to a great error source; establishing an average variability margin may exponentially increase that error.

## Process to Calculate the Emotional Value

**Figure 8:**
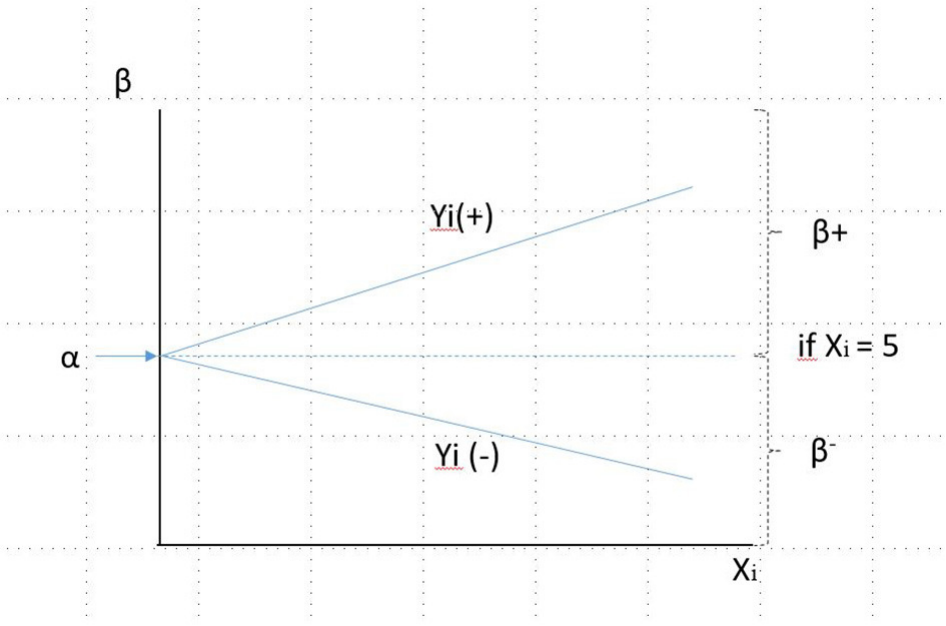
Explanatory regression of the emotional value. Source: own compilation.

Three types of data are needed to transfer the emotional value to monetary units, namely, the price paid, which represents the breakeven price; the scope of the valuation range, which is given by the limits of the fair value; and the degree of satisfaction of the stakeholder in question. According to these three variables, the emotional value can be calculated as a regression where α corresponds to the breakeven point, from which the value that is added or subtracted will be calculated as a product between the distance of the average value and the minimum (B–) or maximum (B+) value multiplied by the degree of satisfaction of the individuals overall. The indicated regression is reflected in Figure 8.


$$\begin{array}{l} \text{Ę}\approx {\text{Y}}_{\text{i}}=\text{α}+\text{E}\\ \text{ E}=\beta {\;}^{*}\;\left({\text{X}}_{\text{i}}-5\right)/10\\ \text{Ę}\approx \text{Yi}=\text{α}+\beta {\;}^{*}\;\left({\text{X}}_{\text{i}}-5\right)/10\\ \text{ Ę}=\text{Integral value}\\ \text{ Ę}=\text{Utility}=\text{Gross emotional value }\left(\text{GEV}\right)\\ \text{ E}=\text{Net emotional value }\left(\text{NEV}\right)\\ \text{ α is the market price }\left(\text{breakeven}\right)\\ \;\beta ={\text{S}}_{\text{i}}{\text{X}}_{\text{i}}\geq 5\to \left[{\text{p}}_{\text{max}}-\text{α}\right];{\text{S}}_{\text{i}}{\text{X}}_{\text{i}}\leq 5\to \left[{\text{p}}_{\text{min}}-\text{ α}\right]\\ {\text{p}}_{\text{max}}\text{ is the upper limit of the fairvalue}\\ {\text{p}}_{\text{min}}\text{ is the lower limit of the fairvalue}\\ {\text{ X}}_{\text{i}}\text{ is the satisfaction score},\;i=\;1-10 \end{array}$$

Before going further into its application, some features of the system should be considered. First, it should be noted that B+ and B– areas do not have to be similar, given that the breakeven price does not have to be located at a midway point between the minimum and maximum price; in other words, the potential value that is added or subtracted is not necessarily symmetrical. Although symmetry is a certain possibility, it is very unlikely to occur. Second, it should be noted that, given that the reasonable value is different for each value transfer variable, the emotional value must be calculated as an aggregate of the value generated by the different variables. This raises the question about the possibility of creating an average beta for the set of variables and using a single weighting regarding satisfaction; this method has been used in some earlier practical work (Ruiz-Roqueñi, 2020). It has the advantage of simplifying the calculation and the data needed, and the clear disadvantage is that it introduces a large error factor. It can be assumed that the error introduced and to what extent it can be pinpointed, in practice, avoided, and whether or not it compensates the ever-increasing complexity of the calculation can be seen, using practical comparisons between the strict methodological application and this simplified one.

We are going to perform a mental experiment using fictitious data to facilitate the understanding of the process to calculate the emotional value.

Variable V_1_ assumption:

The emotional value generated is equal to €3.05, which is obtained using the formula proposed above to the experiment data.

$$\begin{array}{l} \text{E}=\beta {\;}^{*}\;\left({\text{X}}_{\text{i}}-5\right)/10=10{\;}^{*}\;0.305=\;3.05 \end{array}$$

The integrated value of the proposed variable is obtained by adding the emotional value to the market value.

$$\begin{array}{l} {\text{Y}}_{1}=\text{α}+\beta {\;}^{*}\;\left({\text{X}}_{\text{i}}-5\right)/10=15+\left(25-15\right){\;}^{*}\;\left(8.05-5\right)/10\\ \;=15+10{\;}^{*}\;0.305=15+3.05=\;18.05 \end{array}$$

The aggregate value of the set of variables is obtained by adding all the variables that generate value for the benchmark stakeholder.

$$\begin{array}{l} \text{Ę}=\sum \text{Y}\left(1\ldots n\right)={\text{Y}}_{\text{i}} \end{array}$$

**Figure 9:**
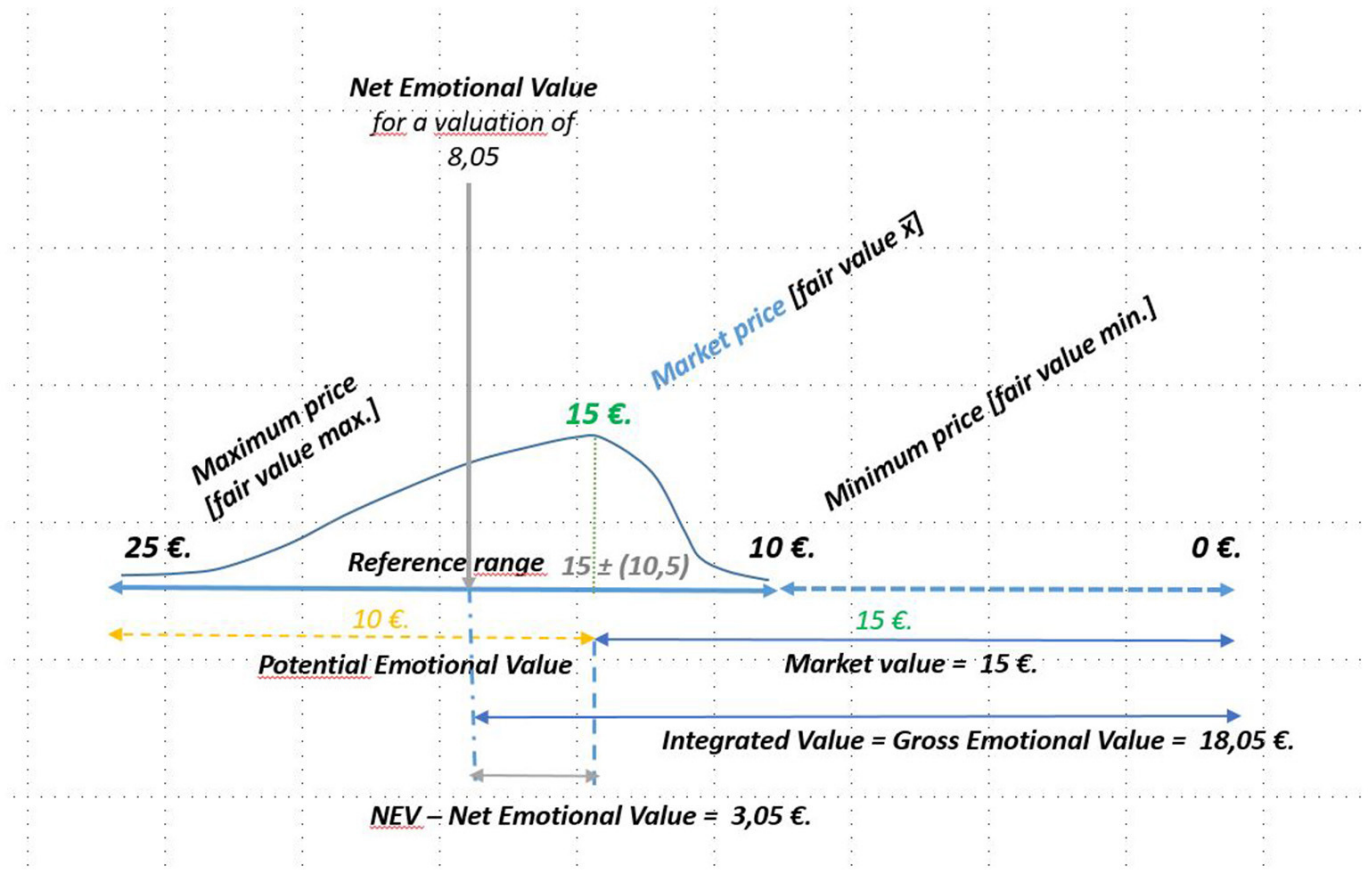
Representation of the NEV. Source: own compilation.

Figure 9 visually reflects the analysis conducted.

## Conclusion and Research Limits

The main contribution of the study consists of identifying the monetary quantification method of the social value that is coherent with the orthodox economic theory so that it not only acts as a means to identify the different components of that value in microeconomic terms but can also be used to conceptually substantiate the definition and the calculation of the emotional value as an added value to the market price. The mathematization of the calculation using a regression allows the result to be objectified, provided that the data referring to the fair value are shared by the analysts. In any event, if that is not so, the problem moves from a confusing field, as has been the case of the conceptualization of the elements comprising the emotional subjective experience allocated to the utility of a good or service up to now, to the estimation of the fair value; an analysis that already has an extensive background in the field of accounting, though it is related to assets to a greater extent than to products or services.

The ability to monetize the emotional value transferred through a set of variables provides better knowledge of the integrated social value [ISV] that an entity transfers to or detracts from society. In addition, the ability to identify the potential emotional value [PEV] allows the percentage that the organization is activating and, therefore, the resulting margin for improvement to be known.

Beyond the sphere of social accounting or the strategic management of the value, the emotional value allows the utility concept to be specified and, therefore, means it can be empirically compared, thus, avoiding the tautological circle in which it is usually immersed.

The limitations include the research is not completely or systematically addressing the calculation of the social value in those cases where there is no market transfer. Although a fair breakeven price can be generally established, it is true that, as there is not a payment or it is subsidized, α is equal to zero, in any event, a lower amount than the breakeven price, which involves a more complex calculation of the transferred emotional value. Despite it being a specific case of the general model, the fact that an important part of the value generated in social accounting may be non-market means progress should be made in the systematization of the calculation in this environment.

Although it is a problem external to the model itself, the correct identification of the fair value of each variable is essential to subsequently calculate the emotional value; furthermore, the ability to identify all the value variables without incorporating false positives is also essential for the holistic calculation of the value transferred by an organization to its stakeholders overall and through them to society.

## Possible Lines of Research

From a practical perspective, the most obvious is the application of the model to analyze the emotional value of different entities, including those with monetary social accounting. As already discussed, there is also the challenge of adapting the model in the specific case where the effective price is subsidized, either in full or partial; the systematic development of this option would allow the benchmarking between market, non-market, and mixed entities to be improved, something that is relatively complex at this time.

As the economic value is the result of the sum of the aggregated value and the emotional value, the hypothesis can be put forward that the non-market value would be incorporated in the emotional value; this hypothesis requires appropriate consideration. If that is the case, it would mean that large corporations, where identifying the non-market value is very costly, could ignore this social accounting step and only work with the emotional and market values.

We have only considered the price from among the costs assumed by the recipient, but the opportunity costs associated with the purchase of the good or service should perhaps be considered for a more holistic approach. There is a whole line of research related to the inclusion of the opportunity costs associated with the transfer processes in social accounting.

Tibor Scitovsky develops the concept of motivation as the reason to search for pleasure that leads to a variation in the level of comfort. Meanwhile, lasting comfort leads to boredom, which drives the individual to make an effort. The individual striving to obtain a good generates pleasure and the individual obtaining it generates comfort. Therefore, the trade-off between pleasure and comfort is created. This reflection is consistent with the modification of the utility perceived over time. Therefore, the emotional value should be seen as a dynamic system that is constantly updated over time. Developing a model able to incorporate the calculation of this constant adaptation process would be an extremely interesting research line.

Another complementary but not less interesting line of research is the analysis of the situations where the emotional value is negative and to study how that affects the continuity in the exchange relationship and what is the trade-off of the exchange concerning the transaction costs.

Finally, given that we as people are incapable of managing information comprehensively and we use heuristic shortcuts in the analysis, and we search for satisfaction rather than maximization in the performance, it would be interesting to research if reputation acts as an enabler, not only in decision making but also in the perceived utility.

## Data Availability Statement

The original contributions presented in the study are included in the article/supplementary material, further inquiries can be directed to the corresponding author/s.

## Author Contributions

JR and LS-J designed the paper, developed the theory and example, written the paper, and reviewed it. All authors contributed to the article and approved the submitted version.

## Funding

Funding for this project was received through a research project called US20/11 from the University of the Basque Country to improve the normalization of social and emotional accounting.

## Conflict of Interest

The authors declare that the research was conducted in the absence of any commercial or financial relationships that could be construed as a potential conflict of interest.

## Publisher's Note

All claims expressed in this article are solely those of the authors and do not necessarily represent those of their affiliated organizations, or those of the publisher, the editors and the reviewers. Any product that may be evaluated in this article, or claim that may be made by its manufacturer, is not guaranteed or endorsed by the publisher.

## References

[B1] Arimany-Serrat N. Tarrats-Pons E. (2021). Integrated social value at universities: a guarantee for public subsidies. Sustainability 13:5975. 10.3390/su13115975

[B2] Ayuso S. Sánchez P. Retolaza J. L. Figueras-Maza M. (2020). Social value analysis: the case of Pompeu Fabra University. Sustain. Account. Manag. Policy J. 11, 233–252. 10.1108/SAMPJ-11-2018-0307

[B3] Babad E. Katz Y. (1991). Wishful thinking—against all odds. J. Appl. Soc. Psychol. 21, 1921–1938. 10.1111/j.1559-1816.1991.tb00514.x

[B4] Barba-Sánchez V. Calderón B. Calderón M. J. Sebastián G. (2021a). Aproximación al valor social de un colegio rural agrupado: el caso del CRA “Sierra de Alcaraz.” CIRIEC España Rev. Econ. Pública Soc. Coop. 101, 85–114. 10.7203/CIRIEC-E.101.18098

[B5] Barba-Sánchez V. Salinero Y. Estevez P. J. (2021b). Monetising the social value of inclusive entrepreneurship: the case of the Abono Café social economy enterprise. CIRIEC-España 101, 115–141. 10.7203/CIRIEC-E.101.18158

[B6] Bernal R. San-Jose L. Retolaza J. L. (2019). Improvement actions for a more social and sustainable public procurement: a Delphi analysis. Sustainability 11:4069. 10.3390/su11154069

[B7] Bernoulli D. (1738). Specimen theoriae novae de mensura sortis. Commentarii Academiae Scientiarum Imperialis Petropolitanae 5, 175–192.

[B8] Blázquez V. Aguado R. Luis Retolaza J. (2020). Science and technology parks: measuring their contribution to society through social accounting. CIRIEC España Rev. Econ. Pública Soc. Coop. 100, 277–306. 10.7203/CIRIEC-E.100.18169

[B9] Etxanobe A. (2020). Marco de referencia para la integración de la contabilidad social en la gestión estratégica de las empresas de economía social. CIRIEC España Rev. Econ. Pública Soc. Coop. 100, 207–237. 10.7203/CIRIEC-E.100.18118

[B10] Freeman E. Retolaza J.L. San-Jose L. (2020). Stakeholder accounting: towards an expanded accounting model. CIRIEC España Rev. Econ. Pública Soc. Coop. 100, 89–114. 10.7203/CIRIEC-E.100.18962

[B11] Guzmán-Pérez B. Mendoza J. P. Monteverde M. V. Roman C. (2018). El valor social de las cofradías de pescadores de Canarias. AECA Revista de la Asociación Española de Contabilidad y Administración de Empresas 124, 18–21.

[B12] Guzmán-Pérez B. Pérez-Monteverde M. V. Mendoza-Jiménez J. Román-Cervantes C. (2021). Social value and urban sustainability in food markets. Front. Psychol. 12:689390. 10.3389/fpsyg.2021.689390 34220652 PMC8241922

[B13] Kahneman D. Tversky A. (1972). Subjective probability: a judgment of representativeness. Cogn. Psychol. 3, 430–454. 10.1016/0010-0285(72)90016-3

[B14] Kahneman D. Tversky A. (1979). Prospect theory: an analysis of decision under risk. Econometrica XLVII, 263–291.

[B15] Lazcano L. San-José L. Retolaza J. L. (2019). Social Accounting in the social economy: a case study of monetizing social value, in Modernizationand Accountability in the Social Economy Sector, eds Ferreira A. Marques R. Azevedo G. Inacio H. Santos C. (Hershey, PA: IGI Global), 132–150. 10.4018/978-1-5225-8482-7.CH008

[B16] Lazkano L. Beraza A. (2019). Social accounting for sustainability: a study in the social economy. Sustainability 11:6894. 10.3390/su11246894

[B17] Lazkano L. Beraza A. San-Jose L. (2020). Determining success factors in the implementation of social accounting. CIRIEC España Rev. Econ. Pública Soc. Coop. 100, 177–205. 10.7203/CIRIEC-E.100.18195

[B18] Mendizabal X. Garcia-Merino D. (2021). Social value measurement in basketball clubs: is it possible? CIRIEC España Rev. Econ. Pública Soc. Coop. 101, 57–83. 10.7203/CIRIEC-E.101.18384

[B19] Mendizabal X. San-Jose L. Garcia-Merino J. D. (2020). Understanding and mapping stakeholders of sport clubs: particularities. Sport Bus. Manag. Int. J. 10, 359–378. 10.1108/SBM-04-2019-0029

[B20] Retolaza J. L. Aguado R. San-Jose L. (2020). Social accounting as an enabling tool to develop collective organizational citizenship behavior in the diocese of Bilbao. Front. Psychol. 11:77. 10.3389/fpsyg.2020.00077 32116904 PMC7033442

[B21] Retolaza J. L. San-Jose L. (2021). Understanding social accounting based on evidence. SAGE Open 11:1–14. 10.1177/21582440211003865

[B22] Retolaza J. L. San-Jose L. Ruiz-Roqueñi M. (2016). Social Accounting for Sustainability Monetizing the Social Value. Cham: Springer. 10.1007/978-3-319-13377-5

[B23] Ruiz-Roqueñii M. (2020). Cuantificación del Valor Emocional. El caso de Unión de Cooperativas Agrarias de Navarra (UCAN). CIRIEC España Rev. Econ. Pública Soc. Coop. 100, 155–175. 10.7203/CIRIEC-E.100.18067

[B24] Samuelson P. Nordhaus W. (1989). Economics, 13 Edn. New-York, NY: McGraw-Hill.

[B25] Samuelson P. A. (1993). Altruism as a problem involving group versus individual selection in economics and biology. Am. Econ. Rev. 83, 143–148.

[B26] San-Jose L. Retolaza J. L. Bernal R. (2019). Social value added index: a proposal for analyzing hospital efficiency. Gaceta Sanitaria 35, 21–27. 10.1016/j.gaceta.2019.08.011 31776045

[B27] Simon H. A. (1972). Theories of bounded rationality. Decis. Org. 1, 161–176.

[B28] Simon H. A. (1979). Rational decision making in business organizations. Am. Econ. Rev. 69, 493–513.

[B29] Tirado-Valencia P. Ayuso S. Fernández-Rodríguez V. (2021). Accounting for emotional value: a review in disability organizations. Front. Psychol. 12:741897. 10.3389/fpsyg.2021.741897 34630255 PMC8497962

[B30] Torres-Pruñonosa J. Raya J. M. Dopeso-Fernández R. (2020). The economic and social value of science and technology parks. The case of tecnocampus. Front. Psychol. 11:632600. 10.3389/fpsyg.2020.632600 33424732 PMC7786403

[B31] Von Neumann J. Morgenstern O. (1947). Theory of Games and Economic Behavior, 2nd rev.

